# Solidified Creosote

**Published:** 1862-07

**Authors:** 


SOLIDIFIED CREOSOTE.—With special reference to its employment in caries of the teeth, Mr. St. Martin has mixed fifteen parts by weight of creosote with ten of collodion, obtaining a mixture of the consistence of jelly, which is used just as if the collodion had not been added.—*Bullet Gén de Thërap., etc.*

